# Plasma Proteome Profiles Associated with Diet-Induced Metabolic Syndrome and the Early Onset of Metabolic Syndrome in a Pig Model

**DOI:** 10.1371/journal.pone.0073087

**Published:** 2013-09-23

**Authors:** Marinus F. W. te Pas, Sietse-Jan Koopmans, Leo Kruijt, Mario P. L. Calus, Mari A. Smits

**Affiliations:** 1 Animal Breeding and Genomics Centre (ABGC), Wageningen UR Livestock Research, Lelystad, The Netherlands; 2 Department of Animal Sciences, Adaptation Physiology Group of Wageningen University, AH Wageningen, The Netherlands; Paris Institute of Technology for Life, Food and Environmental Sciences, France

## Abstract

Obesity and related diabetes are important health threatening multifactorial metabolic diseases and it has been suggested that 25% of all diabetic patients are unaware of their patho-physiological condition. Biomarkers for monitoring and control are available, but early stage predictive biomarkers enabling prevention of these diseases are still lacking. We used the pig as a model to study metabolic disease because humans and pigs share a multitude of metabolic similarities. Diabetes was chemically induced and control and diabetic pigs were either fed a high unsaturated fat (Mediterranean) diet or a high saturated fat/cholesterol/sugar (cafeteria) diet. Physiological parameters related to fat metabolism and diabetes were measured. Diabetic pigs' plasma proteome profiles differed more between the two diets than control pigs plasma proteome profiles. The expression levels of several proteins correlated well with (patho)physiological parameters related to the fat metabolism (cholesterol, VLDL, LDL, NEFA) and diabetes (Glucose) and to the diet fed to the animals. Studying only the control pigs as a model for metabolic syndrome when fed the two diets showed correlations to the same parameters but now more focused on insulin, glucose and abdominal fat depot parameters. We conclude that proteomic profiles can be used as a biomarker to identify pigs with developing metabolic syndrome (prediabetes) and diabetes when fed a cafeteria diet. It could be developed into a potential biomarkers for the early recognition of metabolic diseases.

## Introduction

In the modern western world with its high food availability and preference for food with high saturated fat content, the number of people showing signs of obesity and diabetes is increasing. Obesity is characterized by increased fat storage in fat depots of the body. However, fat depots differ, e.g. in gene expression regulation [1]. In itself stored fats are not detrimental to health. Fat storage is even required for proper functioning of the adipocytes producing leptin for regulation of satiety and adipo-chemokines for regulation of immune function [2], [3], [4]. Furthermore, fatty acids have important cellular functions including gene regulation for energy requiring processes, energy expenditure regulation, and as components of cell membranes and diverse proteins. However, obesity may be a health threatening condition due to its relationship with other metabolic syndromes, including diabetes due to insulin resistance or insulin irresponsiveness [5], increased blood cholesterol levels inducing atherosclerosis and related problems of heart failure and high blood pressure. Nutrient sensors play an important role in the regulation of energy homeostasis [6], [7]. Deregulation of energy homeostasis, may lead to obesity and other metabolic syndromes.

The fully developed phenotypes of metabolic diseases can be measured easily and tools have been developed for monitoring and controlling the development of metabolic diseases – e.g. the blood glucose level of diabetic patients is used as a biomarker to monitor the status of insulin function and to control medication. However, due to the interaction between the genotype and storage of fatty acids related to obesity, the development of these health threatening conditions is unpredictable, and early signs of the development of the metabolic diseases are not easily recognized. As a result approximately 25% of the (pre)diabetes patients are unaware of their physiological condition. Instead of monitoring the diseases and treating the consequences of the metabolic syndromes, detection of the first signs of the development of these diseases could help to take preventive actions and interfere with the full development of the disease. Therefore, a new generation of biomarkers, enabling the detection of the first signs of metabolic changes, need to be developed. Preferentially, such biomarkers need to be measurable in easily accessible body fluids such as urine, saliva or blood. The application of –“genome-wide” technologies, such as transcriptomics, proteomics and metabolomics, offers exciting opportunities to discover such predictive biomarkers for metabolic diseases [8], [9], [10], [11]. In this paper we applied proteomics as a high throughput methodology enabling the screening of proteome expression profiles of blood plasma.

We have taken a first approach for the development of such plasma-protein proteomics-based biomarkers using a validated pig model for metabolic syndrome and diabetes [12], [13]. It is known that a disbalance in fat metabolism and storage can be induced by offering cafeteria food, rich in saturated fats to pig selected for leanness for several decades [14], [15]. Furthermore, physiologically, pigs and humans show many similarities [16]. The advantage of the use of a pig model is that metabolic syndrome can be experimentally induced, that the experimental units can be kept under controlled environmental and dietary conditions, and that during all developmental stages different biological samples can be collected for deep phenotyping. Therefore, pigs are a good model system for studying human metabolic diseases in a time-dependent manner. Previously, Bell et al. [17] reported on the use of proteomic approaches for the analysis of serum protein profiles in extremely fat miniature swine. We have used the model for metabolic syndrome and diabetes in Yorkshire x Landrace pigs for the detection of a new generation of biomarkers associated with the first signs of metabolic changes related to metabolic syndromes like diabetes. The objective of this investigation was to develop proteomics profiles differentiating healthy pigs and pigs with signs of early onset of metabolic syndromes, and with related insulin resistance metabolic syndromes. The results indicate that several peaks in the plasma proteome profile can be used as biomarkers to detect the early onset of metabolic syndrome.

## Materials and Methods

### Animals and experimental development of obese and diabetes metabolic syndromes

The performed research is in compliance with the ARRIVE guidelines on animal research [18]. Experimental protocols describing the management, surgical procedures, and animal care were reviewed and approved by the ASG-Lelystad Animal Care and Use Committee (Lelystad, The Netherlands).

Twenty-four 11 weeks old (approximately 30 kg) male Yorkshire x Landrace pigs were divided in a control (N = 11) and an experimental (N = 13) group. The experimental group (N = 13) received Streptozotocin that induces diabetes by destroying the B-cells in the pancreas [19], [20], [21], [22]. The diabetic pigs were checked for blood glucose levels higher than 10 (data not shown) [12], [13]. Approximately half of the animals in both groups (N = 5 for control pigs and N = 6 for Streptozotocin treated experimental pigs) received a “cafeteria diet” consisting of high saturated fat, half of the animals (N = 6 for control pigs and N = 7 for Streptozotocin treated experimental pigs) received a “Mediterranean diet” consisting of high unsaturated fat for ten weeks. Table 1 describes the composition of the feeds. Control and diabetic pigs were fed 2 meals per day (from 08:00 till 09:00 h and from 15:00 till 16:00) during which the pigs had ad libitum access to food with a follow-up time of 10 weeks. Average daily feed intake was similar in both control pig groups and the diabetic pigs fed the Mediterranean diet (23±2 vs 22±1 vs 24±2 MJ/day) but higher in the diabetic pigs fed the cafeteria diet (31±3 MJ/day, p<0.03 respectively). The afternoon meal, preceding the collection of 16 hours fasting plasma samples was restricted and identical for all pigs (meal size was 6 MJ). Development of obesity and diabetes was checked as described previously [12], [13]. A number of physiological parameters related to body weight gain, body composition, fatness metabolism, and diabetes were measured (Table 2) (file S1), in order to determine the physiological effects of the different treatments and feeds given to the animals. Physiological parameters were measured either 3 hours postprandial or after 16 hours fasting as indicated in the Tables.

**Table 1:** Composition of the high unsaturated fat (Mediterranean) diet and the high saturated fat (cafeteria) diet.

Feed components	Mediterranean Diet	Cafeteria Diet
Soy beans, extracted	51.3	51.3
Potato protein	50	50
Wheat gluten meal	118.3	118.3
Cellulose	100	100
Sucrose	100	100
Fructose	150	150
Native Pea starch	150	150
Animal fat (lard)	0	250
Trisun 80, high oleic sunflower oil	55.9	0
Canola	142.5	0
Cornoil	45	0
Fishoil	6.6	0
Cholesterol (extra)	0	10
Chalk marl	11	11
Monocalcium phosphate	9.9	9.9
Sodium chloride	4.7	4.7
Mineral/vitamin premix^a^	2	2
L-Lysine hydrochloride	2.8	2.8
**Total (g/kg)**	**1000**	**1010**
**Gross Energy (GE; Mj/kg)**	**23.4**	**23.4**

a: This vitamin and trace mineral premix contained per kg diet: vitamin A (retinol) - 1750 IU; vitamin D3 (cholecalciferol) - 200 IU; vitamin E (tocopherol) - 11 IU; vit. K1 (phylloquinone) - 0.5 mg; vitamin B1 (thiamin) - 1.0 mg; vitamin B2 (riboflavin) - 4 mg; d-pantothenic acid - 9 mg; niacin (vitamin B3, nicotinic acid) - 12.5 mg (available); biotin (vitamin H) - 50 µg; vitamin B12 (cyanocobalamin) - 15 µg; folic acid (folacin) - 0.3 mg; vit. B6 (pyridoxin) - 1.5 mg; choline - 400 mg; Fe - 80 mg; Zn - 54 mg; Mn - 30 mg; Co - 0.15 mg; I - 0.14 mg; Se - 0.25 mg; antioxidants (E310,320,321) - 50 mg; with maize starch as carrier.

**Table 2:** Plasma parameters after 16

Treatment group	Control				Diabetic				diet	F-prob	
Diet	Mediterranean		Cafeteria		Mediterranean		Cafeteria				
	Mean	SEM	Mean	SEM	Mean	SEM	Mean	SEM	lsd	diet	diab
Plasma glucose (mM)	5.8	0.1	5.7	0.2	13.5	1.4	17.2	1.2	2	0.06	<0.001
Plasma Insulin (ng/mL)	0.08	0.016	0.101	0.024	0.053	0.01	0.073	0.018	0.031	0.2	0.08
Plasma triglyceride (mM)	0.16	0.02	0.15	0.02	0.42	0.14	0.36	0.16	0.2	0.72	0.04
CRP (mg/L)	16	5	16	1	14	2	21	2	5.4	0.24	0.65
NEFA (mM)	0.38	0.05	0.34	0.07	0.5	0.14	0.72	0.16	0.23	0.38	0.04
Cholesterol (mM)	2.3a	0.4	4.8a	0.7	2.2a	0.2	15.0b	3.7	3.6	<0.001	0.01
LDL (mM)	1.2a	0.3	2.2a	0.2	1.0a	0.1	5.4b	0.9	1	0.01	<0.001
HDL (mM)	0.6	0.1	0.8	0.1	0.8	0.1	1.3	0.2	0.3	0.01	0.03
VLDL (mM)	0.5a	0.1	1.8a	0.4	0.4a	0	8.3b	2.6	2.8	0.001	0.03
HDL/LDL (%)	0.53	0.13	0.41	0.08	0.85	0.14	0.28	0.07	0.24	0.005	0.36
HDL/Cholesterol (%)	26bc	4	19ab	4	36c	4	11a	3	12	<0.001	0.69

The standard errors of the means (SEM), the least square differences (lsd) and p-value (F-prob) are tabulated. tabulated. Different letters a, b or c in the same row differ statistically at p<0.05. Nb. interactions for cholesterol, LDL, VDL and HDL/Cholesterol (lsd is here diet*diabetes); F prob HDL/LDL = 0.07; lsd = 0.35.

### Sample preparation

In the eighth week blood samples were collected 3 hours postprandial and after 16 hours of fasting and physiological parameters were determined. At 9 weeks blood samples were collected after 16 hours of fasting and used for plasma proteome determination, and after 10 weeks the body composition parameters were determined at slaugher. The physiological parameters were analyzed as described before [12], [13]. In short: Blood samples collected in heparinized (150 USP. U. Lithium Heparin, 10 mL Venoject, Terumo, Leuven, Belgium) or EDTA (ethylenediaminetetraacetic acid, (0.47 mol/L EDTA, 10 mL Venoject, Terumo, Leuven, Belgium) tubes were immediately chilled at 0°C on water with ice, and centrifuged at 4°C for 10 minutes at 3000 rpm. Plasma aliquots were stored at -80°C for later analyses.

Plasma glucose was analyzed with the Glucose liquiUV mono kit (Human, Wiesbaden, Germany), plasma nonesterified fatty acids were analyzed with the WAKO kit (Neuss, Germany) and plasma and tissue triglycerides with a kit from Human (Wiesbaden, Germany). Total, LDL and HDL cholesterol concentrations in plasma were determined with liquicolor kits (Human, Wiesbaden, Germany) and VLDL

cholesterol was calculated as total cholesterol minus LDL and HDL cholesterol. Plasma insulin concentration was measured using a Delfia assay (test kit by Perkin Elmer Life Sciences Trust by Wallac Oy, Turku, Finland). This specific pig insulin assay was validated using pig insulin standards, as indicated before [23].

Plasma C-reactive protein (CRP) was analyzed with a kit (CRP-hs, Human, Wiesbaden, Germany).

To detect the proteins present in low levels in plasma, we used ProteoMiner® (BioRad, Veenendaal, The Netherlands) performed according to manufacturer's recommendation. Briefly, 525 mg Bulk Beads were swelled by rehydration with 10 ml 20% (v/v) aqueous EtOH. One hundred µl of this beads solution was washed with water and PBS in a 96-well filter plate (Pall-5039) and subsequently mixed with 200 µl centrifuged EDTA plasma for two hours at 4°C. After binding and three times washing the beads with 200 µl PBS, the proteins were eluted three times with 20 µl of each of the four elution reagents; fraction 1 (1 M NaCl, 20 mM HEPES pH 7.5), fraction 2 (200 mM glycine pH 2.4), fraction 3 (60% ethylene glycol), and fraction 4 (33% isopropyl alcohol, 16.7% acetonitrile, 0.1% TFA). Between every elution the beads were mixed for 5 minutes at room temperature, and centrifuged 1 min at 1000 g to collect the eluent from filter plate to a non-binding collection plate (Greiner Bio-one 655900).

### Proteomics

To fractionate the proteome isolates we used protein array types H50 (a hydrophobic protein array type for lipoprotein binding with 10% acetonitrile, 0.1% trifluoroacetic acid (TFA) binding buffer), IMAC (a copper containing protein array type for phosphoprotein binding with 0.1 M sodium phosphate, 0.5 M NaCl pH 7 binding buffer), and CM10 (a weak cation exchanger with low stringent (100 mM sodium-acetate pH 4) and high stringent (100 mM sodium-acetate pH 7) binding buffers for binding of electrically charged proteins). The different ProteinChip arrays were equilibrated with the respective binding buffers. One hundred µl of the 10-fold diluted fractions, in appropriate binding buffer depending of the array (total volume 250 µl), were incubated on the ProteinChip arrays. After 60 minutes incubation with continuous shaking the arrays were washed three times with 200 µl appropriate binding buffer, followed by a wash with 200 µl MilliQ water. After air drying at room temperature, 2×1 µl of a saturated solution of energy absorbing matrix sinapinic acid (SPA) in 50% acetonitrile (v/v), 0.5% trifluoroacetic acid (v/v) was added.

Mass analysis of the bound proteins was performed using the SELDI-TOF (Surface-enhanced laser desorption/ionization-time of flight mass spectrometry) (BioRad) technology with the Protein Chip System Series 4000 (BioRad) equipment in positive operating mode. Mass spectra were collected by the accumulation of laser shots at a laser energy of 3000 n J. Proteins were detected by the m/z values of their peaks. The highest mass to acquire was set at 200,000 kDa; Focus Mass setting was 10,000 kDa. The instrument was calibrated by using the All-in-one ProteinStandard II (BioRad). The mass spectra of the proteins captured on the chips were recorded with the PCS4000 ProteinChip array reader (BioRad) by using ProteinChip Data Manager software 3.5.0. [24].

### Statistical evaluation

The diet-related physiological traits of all four pig groups were analyzed using ANOVA. Post-hoc comparison between groups was done by Student-t-test. The proteomics data were analyzed using Lucid Proteomics Software v2.0 (Bio-Rad Laboratories). The results were evaluated for differential expression between all four groups of pigs. The software provided for the statistical evaluation tools. Significance levels were set to P<0.005. Correlations between peak expression levels and metabolic parameters were determined with the Spearman correlation test. Multivariate data analysis (PCA) were performed using the Lucid Proteomics Software v2.0. Next, the residuals for SELDI-TOF peak patterns and physiological parameters were determined using ANOVA with a Randomized Block Design with diabetes and feed composition as independent effects.

## Results

### Phenotyping of the control and diabetic pigs fed the Mediterranean or cafeteria diet

Seventy different biochemical and other parameters were measured in blood and several organs to determine the (metabolic) reactions of the control and diabetic pigs, and to determine their adaptive metabolic reactions to the two different feed formulations. The major parameters that differ significantly between the four experimental groups are indicated in Table 2. In general, many metabolic parameters differed between the two diets (for detail information see file S1), although diabetic pigs differed more between the two diets than control pigs. The control pigs and diabetic pigs showed many similarities in their response towards the Mediterranean diet, while the two groups showed more different reactions to the cafeteria diet.

### Proteomics profiles differentiating control and diabetic pigs fed the Mediterranean or cafeteria diet

We observed a total of 984 protein peaks in the four fractions of the three different protein arrays. Table 3 shows the number of protein peaks with significant quantitative differences between the groups of pigs in all four fractions for each of the protein arrays. The details of all peaks are given in the file S2. Between the experimental groups significant quantitative differences in specific protein peaks were found with each protein-array -type and testing condition. The number of peaks was approximately similar for all protein arrays and testing conditions. The number of peaks per testing conditions varied between six and 29.

**Table 3:** Plasma proteome profiles comparisons.

	CM10, pH 7	CM10, pH 4	H50	IMAC
Fraction 1	7	11	22	6
Fraction 2	11	20	17	13
Fraction 3	29	25	11	18
Fraction 4	9	12	6	14
Total	56	58	56	51

Number of protein peaks for each of the protein arrays showing differential expression between the four groups of pigs. It should be noted that some of the peaks may be similar in subsequent fractions.

While almost all parameters showed correlations to peak expression levels of 0.5 or more, only ten parameters showed correlations with peak expression levels higher than 0.85. These include the parameter measurements of NEFA, triglycerides, glucose levels, and cholesterol, LDL and VLDL. Only the latter three showed correlations higher than 0.9, up to 0.98 (Table 4). These high correlation values indicate a high similarity in the protein-peak pattern between the cholesterol, LDL and VLDL parameters, suggesting an overlap of the molecular background of these parameters. The ANOVA model with diabetes and feed effects explained 27% of the variance (data not shown). Using the residuals, i.e. the effects corrected for diabetes and feed effects, the correlations between SELDI-TOF peaks and physiological parameters were again determined (Table 4). The results indicate that most correlations remained similar to the original data. The number of correlating peaks were less for some of the results (Table 4). All correlation values are presented in file S3.

**Table 4:** Correlations between plasma proteome profiles and physiological parameters.

Parameter	N peak correlation	Residuals	Peak sharing
	>0.85 (>0.9)		
**Cholesterol during fasting**	60 (25)	26 (18)	all >0.9; most 0.85–0.9
**VLDL during fasting ^a^**	50 (27)	29 (21)	all >0.9; most 0.85–0.9
**LDL during fasting ^b^**	55 (26)	23 (7)	all >0.9; most 0.85–0.9
**HDL/LDL**		1	
**Triglycerids (TG) during fasting ^c^**	1		NEFA postprandial
**TG postprandial**	1		Glucose
**TG in Kidney**	1	1	
**NEFA during fasting ^d^**	1	1	
**NEFA postprandial**	2		TG
**Glucose during fasting**	1		
**Glucose postprandial**	2		TG postprandial

Number of peaks showing correlation >0.85 (>0.9) between protein peak expression levels and physiological parameters in control and diabetic animals fed with the Mediterranean or cafeteria diets. The parameters were measured either 3 hours postprandial or after 16 hours fasting, where indicated. For information on the peak m/z values see file S3. The information after calculating the residuals using a model including effects of diabetes and feed is included. Information about peak similarities is given where appropriate. a: Very Low Density Lipoproteins; b: Low Density Lipoproteins; c: Triglycerides; d: Non Esterified Fatty Acids.

**Figure 1:**
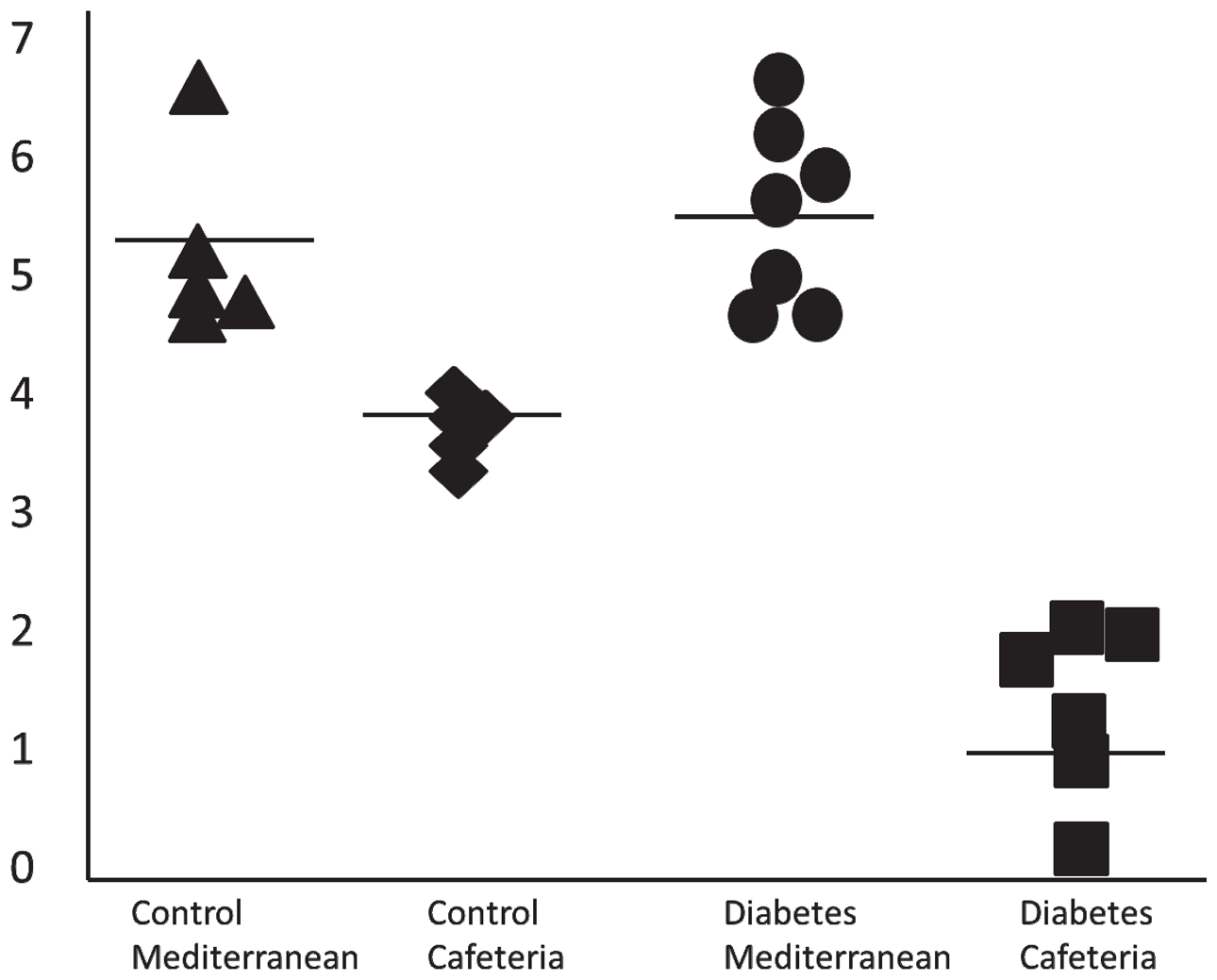
Example of proteome profile differences between control and diabetic pigs fed two diets. Control and diabetic pigs were either fed a healthy Mediterranean diet or a cafeteria diet differing mainly in fatty acid composition. Plasma protein profiles were determined with a SELDI-TOF equipment and analyzed in relation to physiological parameters. Expression levels of peak M/Z 28537, CM10 pH 7, fraction 3. Each dot represent the expression level found in a pig. The horizontal bar represents the mean. ▴: Control Mediterranean diet, ♦: Control Cafeteria diet, •: Diabetes Mediterranean diet, ▪: Diabetes cafeteria diet.

Individual protein peaks differed for their expression levels between the experimental groups. Figure 1 shows a representative example for the expression levels of a peak with a molecular weight of 28537 Da in the four groups of pigs. The results showed that in both the control and diabetic animals given the Mediterranean diet the expression level of the protein was similar with little variation between the animals. Cafeteria feed reduced the expression level in both the control and diabetic pigs, but the diabetic pigs showed larger physiological reaction effects than the control pigs. Similar effects were seen for other protein peaks although the specific effects differed between peaks. Other protein peaks indicated differential expression between control and diabetic pigs fed the Mediterranean diet. The data for all peaks can be found in file S4.

**Figure 2:**
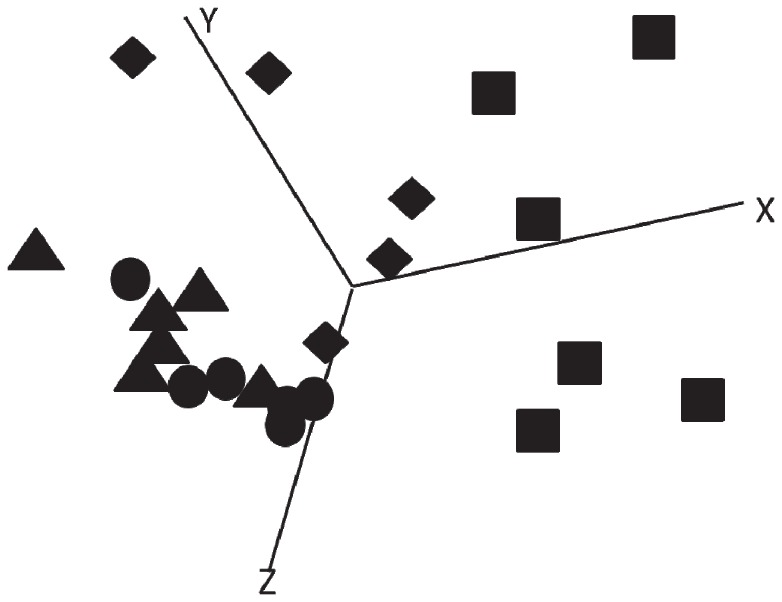
Principal component analysis. Control and diabetic pigs were either fed a healthy Mediterranean diet or a cafeteria diet differing mainly in fatty acid composition. Plasma protein profiles were determined with a SELDI-TOF equipment and analyzed in relation to physiological parameters. Example of the principal component analysis of peaks showing differential expression between the groups of animals: peak M/Z 6623, CM10, pH 7, fraction 3; ▴: Control Mediterranean diet, ♦: Control Cafeteria diet, •: Diabetes Mediterranean diet, ▪: Diabetes cafeteria diet.

Principal component analysis (PCA) was used to further separate the groups based upon the results of the expression levels of a peak in all groups of pigs. Figure 2 shows an example of a PCA. The control and diabetic induced Mediterranean diet groups are highly similar. The cafeteria fed groups differed clearly from each other. The difference between the control group fed the two diets is smaller than the difference between the diabetic pigs fed the two diets.

### Dietary effects in control pigs

PCA analysis of the four experimental groups clearly indicated significant effects of saturated feeds in the diabetic pigs on cholesterol, LDL, and VLDL parameters and their correlated protein peaks. Additional peaks with high correlations were found for glucose/insulin related parameters and for body weight (Table 5). Surprisingly, two-group comparison comparing the Mediterranean and cafeteria dietary effects in the control pigs showed that the saturated fat feed affected the expression of protein peaks related to the cholesterol, LDL, and VLDL groups, although to lower levels compared to the four – group analysis. Comparing the dietary effects in the two-group and four-group analysis showed that three peaks appear in both peak lists (file S3). Although significant, these three peaks did not reflect the highest correlations for the cholesterol, LDL, and VLDL parameters. Several other correlations were found for several organ fatness traits. The residuals, i.e. the effects corrected for diabetes and feed effects, were used to determine the correlations between SELDI-TOF peaks and physiological parameters again (Table 5). The results show that most of the correlations remained similar, while the correlations related to the cholesterol and triglyceride concentrations and plasma transport particles showed increased numbers of correlating peaks. All obtained correlations are indicated in file S3.

**Table 5:** Correlations between plasma proteome profiles and physiological parameters in control pigs fed the two diets.

Parameter	Number of peaks	Residuals
Body weight at slaughter	4	2
Insulin postprandial	4	
Glucose postprandial	3	6
Glucose during fasting	1	1
NEFA^a^ postprandial	2	
NEFA during fasting		17
Cholesterol during fasting (includes VLDL and LDL)	1	15
VLDL^b^	2	21
LDL^c^	3	9
HDL^d^		1
TG^e^ postprandial		6
TG during fasting		1
Retroperitoneal fat (g)	1	
Abdominal fat depot (i.e. Omental fat) (g)	2	
Retroperitoneal fat depot per kg body weight	1	
Omental fat depot per kg body fat	2	

Number of peaks showing correlation >0.9 between protein peak expression levels and physiological parameters in control animals fed with the Mediterranean or cafeteria diets.a: Non Esterified Fatty Acids; b: Very Low Density Lipoproteins; c: Low Density Lipoproteins; d: High Density Lipoproteins; e: Triglycerides.

## Discussion

### The development of biomarkers for the early onset of metabolic disease status without clinical signs

Biomarkers indicate the presence of a biological process, which could be linked to the clinical manifestation of a disease [25]. In our study we assumed that also the metabolic changes related to metabolic diseases could be detected with biomarkers. Liotta et al. [26] suggested that the blood reflects the ongoing physiological state of all tissues. This could be both molecules excreted from the cells (e.g. for signaling to other cells or as “waste”), or via leakage of cells. Taken together, it may be expected that plasma protein biomarkers reflect the metabolic status of an animal or human. Thus, it was reasonable to assume that a changed metabolic status was also reflected in the blood plasma. Similarly, Rao et al. [10] showed salivary proteome profile differences related to diabetes in human. Furthermore, Doherty et al. [8] suggested that the symptoms of many diseases may take a considerable period of time to manifest. Thus, it may be expected that the molecular signatures of the disease could be measurable long before the clinical manifestation. This makes it even more important to be able to detect the early onset changes in a metabolic disease pathway. Biomarkers enabling to detection of the early onset of the metabolic disease state could be used both for prevention of the disease, or as a target for treatment. The proteomics research in relation to diabetes has been reviewed by Sundsten and Ortsäter [11]. Proteomic profiles have been produced of pancreatic islets, β-cells, adipose tissue, muscle tissue, liver tissue, and blood serum. However, all these proteome profiles represent differences between healthy and fully diabetes metabolic status. No research into the early onset disease status – where no clinical signs of the disease can be observed yet - in relation to the healthy status have been reported. It should be interesting to compare the proteome profile differences in metabolic status between early onset and fully developed metabolic disease status in relation to healthy status. The question is whether the observed differences all direct into similar directions as the observed effects in the fully developed metabolic disease.

### Pig model for metabolic syndromes studies

The biological contrast in the current experimental design was created in a two-step procedure. In the first step chemical treatment was used to induce diabetes in half of the experimental pigs [12], [13], [23]. This creates an absolute contrast to the not-diabetic control pigs. In the second step, feed formulations - mainly differing in fat composition (unsaturated vs. saturated) - were used to create biological variation within the diabetic as well as in the control animal groups. The latter created a high saturated fat-metabolism effect in both control and diabetic pigs, although the effects differed between the two groups of animals. The influence of the diets used in both control pigs and diabetes induced pigs highlights the effect of feed composition on control and diabetic status. The experimental design and the observed physiological and metabolic correlations allow to find biomarkers for the detection of early and late metabolic disease effects, respectively related to diet composition.

The diabetic pigs showed the typical biochemical changes related to the metabolic disorder. In this study we showed that this is accompanied by plasma proteome profile changes, indicating a relationship between the plasma proteome profile changes and the metabolic disorder. Furthermore, related changes in the reaction of the metabolic disorder to diet composition were also accompanied by changes in the plasma proteome profiles. Thus, these proteome peak profiles can be regarded as potential new biomarkers for the clinical diabetes disease. More importantly, also in the non-diabetic control pigs we observed diet-related changes in the proteome profiles related to the LDL, VLDL and cholesterol levels in blood, which can be regarded as potential early metabolic syndrome indicators of the early stages of the metabolic syndrome induced by diet.

### Proteome profiles differentiating control and diabetic metabolic syndrome pigs

The expression levels of proteomics peaks correlated with parameters relating specifically to cholesterol and fatty acid blood plasma levels, and fatty acid transport vehicles. These results showed that proteomics can potentially be used to detect the diabetic metabolic disease status of pigs. Especially the high correlations found for the cholesterol levels, and the LDL and VLDL particles are interesting. Identification of the correlated protein peaks should reveal whether they are constituents of the protein cores of the particles or whether they are proteins with thus far unknown functions in the metabolic syndrome. The finding of biomarkers relating to fatty acid metabolism may not be surprising since adipose tissue biomarkers have been observed both for obesity and type-2 diabetes [25].

The high number of protein peaks (up to 60) suggest that more proteins contribute to the associations. However, it should be noted that 60 peaks not necessarily indicate 60 different proteins since there may be some overlap of peaks between different fractions and array types, and degradation products of proteins will be visible as independent peaks. On the other hand, we have no indication that (widespread) protein degradation occurred in our samples. The peaks may be easily accessible in blood plasma for biomarker test development.

The here reported protein peaks show differential expression in at least one of the four groups of pigs and may therefore be potential biomarkers to detect early onset of the metabolic syndrome. Two main differences were found: (1) peaks showing differential expression between control and diabetic induced pigs fed with the Mediterranean diet, and (2) peaks showing differential expression between control and diabetic induced pigs fed with the cafeteria feed, with the latter group showing larger differences in peak expression levels. This may indicate that diabetic pigs are extremely sensitive to differences in feed composition. These pigs may be metabolically less efficient for fat metabolism as suggested by the correlations with a number of protein peaks relating to fatty acid levels in the blood. The finding of potential biomarkers showing only differential expression for the diabetic cafeteria fed pigs as compared to all other groups of pigs may indicate that especially diabetes pigs are vulnerable to physiological imbalanced reactions to the unhealthy high saturated fat cafeteria feed. This indicates that this group showed major differences in metabolism as compared to the other groups. We conclude that this relates to the diabetic status of the pigs.

### Dietary effects in non-diabetic control pigs

In the chemically-induced-diabetes pigs the early stages of obesity and diabetes cannot be studied. By excluding these samples from the analysis we studied the early effects of saturated fats and our results indicated that we studies in fact the early stages of diet induced metabolic syndrome. Metabolic syndrome, and ultimately overt diabetes may result from long time intake of an unhealthy high saturated fat diet. In these analyses the correlations to glucose/insulin/NEFA/cholesterol/related parameters were found, next to body weight and abdominal fat depots. These correlations suggest that the diet indeed induced early stages of metabolic syndrome. But the blood glucose levels showed that these pigs were not diabetic (yet), so this suggest that we have here the early signs of developing metabolic syndrome, e.g. diabetes. The correlated protein peaks may be valuable indicators of early onset of diabetes and could be useful for the development of biomarkers to detect early onset of diabetes. The observed correlations with the variation in the physiological parameters suggested that these proteins may be early indicators of the aberrations form the normal situation of the metabolic status of the animals. Furthermore, the correlations with the body weight and fatness parameters suggest that developing obesity/excessive fatness may be the causative mechanism although the animals were not obese.

Only three peaks were shared by the 4-group and 2-group analyses. This suggests that most correlations found using all animals in the analysis were related to effects of full blown diabetes, probably because of the large contrast created by the treatment. Thus, these three peaks may specifically represent diet-induced effects in diabetic animals and may relate to the clinical stage of the disease. While these results clearly indicate that the proteome of the blood plasma was changed considerably by the diet and the metabolic syndrome, such parameters may be less important to detect and monitor early onset of metabolic syndrome. Therefore, it may be more interesting to focus on the protein peaks with correlations to glucose/insulin parameters in the analysis of the control pigs.

### The road towards early recognition of the development of human metabolic syndromes

We investigated the proteome of plasma for biomarkers that could be used for the clinical and preclinical early detection of diabetes and feed composition-related metabolic changes in pigs. We concluded that proteomic biomarkers can be identified in the plasma of pigs that are related to 1) clinical disease, 2) traditional biochemical measurements in diabetic cases, 3) biomarkers in clinically healthy individuals that develop the first physiologic signs of a metabolic syndrome, e.g. diabetes, [6]) subset of latter biomarkers overlap with protein biomarkers of clinical cases. Thus, identified proteins in control animals fed the saturated fat diet may be potential biomarkers to detect early onset of metabolic syndrome or diabetic disease in humans used to identify pigs with metabolic syndrome (prediabetes) and diabetes when fed a cafeteria diet. In addition, the proteomic biomarkers can be used to screen for healthy and unhealthy diets in control and diabetic pigs, and perhaps in humans too, especially in predestined individuals. The results of the ANOVA model showed that only limited effects of the diabetes experiment and feed composition were observed. Thus, most of the variation explained by the correlations related to the metabolic syndrome effects. The improved results for the control (i.e. not diabetic) animals only suggest that these peaks indeed may be early indicators of the metabolic syndrome development due to the feed composition in the cafeteria diet fed pigs. This suggests that these biomarkers can also be used to differentiate healthy and potentially metabolic syndrome developing pigs. Expressional changes can be seen in these biomarkers during the early stages of the onset of the metabolic disease in these pigs. Such biomarkers, if similar to human biomarkers, could be easily tested in clinical studies to predict the metabolic status of individuals who may be in the process of developing diabetes or obesity-related metabolic diseases. Since humans and pigs show many physiological similarities it may be expected that these proteomic biomarkers are predictive in humans too [14], [26]. These biomarkers may be helpful in early detection of pre-diabetes in not-yet-ill individuals. As a next step towards further development of biomarkers the proteins of the peaks should be identified. This step need to be followed by validation of the proteins using an independent group of pigs before studies with human derived material can be initiated.

## Supporting Information

File S1 **Biochemical parameters of the control and chemically-induced diabetes pigs fed the Mediterranean and cafeteria feeds.**
(XLSX)

File S2 **Details of all protein peaks showing differential expression related to control and diabetic pigs fed either Mediterranean diet or a cafeteria diet in both pig models.**
(XLSX)

File S3 **All correlations between all protein peaks and all physiological parameters.**
(XLSX)

File S4 **Expression diagrams of all peaks showing differential expression related to control and diabetic pigs fed either Mediterranean diet or a cafeteria diet in both pig models.**
(PPTX)
